# ‘Root of all success’: Plasticity in root architecture of invasive wild radish for adaptive benefit

**DOI:** 10.3389/fpls.2022.1035089

**Published:** 2022-11-16

**Authors:** Samik Bhattacharya, Franziska Gröne, Felix Przesdzink, Jotham Ziffer-Berger, Oz Barazani, Klaus Mummenhoff, Niels Kappert

**Affiliations:** ^1^ Department of Biology, Botany, Osnabrück University, Osnabrück, Germany; ^2^ Department of Biology, Levinsky College of Education, Tel-Aviv, Israel; ^3^ Herbarium, Steinhardt Museum of Natural History, Tel-Aviv, Israel; ^4^ Institute of Plant Sciences, Agricultural Research Organization, Rishon LeZion, Israel

**Keywords:** root system architecture (RSA), root plasticity, Raphanus, East Mediterranean, soil surrogates, adaption, habitat preference

## Abstract

Successful plant establishment in a particular environment depends on the root architecture of the seedlings and the extent of edaphic resource utilization. However, diverse habitats often pose a predicament on the suitability of the fundamental root structure of a species that evolved over a long period. We hypothesized that the plasticity in the genetically controlled root architecture in variable habitats provides an adaptive advantage to worldwide-distributed wild radish (*Raphanus raphanistrum*, *Rr*) over its close relative (*R. pugioniformis*, *Rp*) that remained endemic to the East Mediterranean region. To test the hypothesis, we performed a reciprocal comparative analysis between the two species, growing in a common garden experiment on their native soils (Hamra/Sandy for *Rr*, Terra Rossa for *Rp*) and complementary controlled experiments mimicking the major soil compositions. Additionally, we analyzed the root growth kinetics *via* semi-automated digital profiling and compared the architecture between *Rr* and *Rp*. In both experiments, the primary roots of *Rr* were significantly longer, developed fewer lateral roots, and showed slower growth kinetics than *Rp*. Multivariate analyses of seven significant root architecture variables revealed that *Rr* could successfully adapt to different surrogate growth conditions by only modulating their main root length and number of lateral roots. In contrast, *Rp* needs to modify several other root parameters, which are very resource-intensive, to grow on non-native soil. Altogether the findings suggest an evo-devo adaptive advantage for *Rr* as it can potentially establish in various habitats with the minimal tweak of key root parameters, hence allocating resources for other developmental requirements.

## Introduction

The journey of survival and success of the new generation of plants starts after the radicle emerge from the germinating seed to seek water and nutrient and finally anchor themselves to the habitat. However, the architecture of the root system is paramount to successfully establishing the seedlings within highly competitive ecosystems (Malamy, 2005; Lynch, 2018), as there is limited resource opportunity and tight genetic control of the root structure (Lynch, 1995; Lynch, 2018). Therefore, a seedling must grow a robust root system to accumulate resources to survive competitors and defend against biotic and abiotic stresses (Comas et al., 2013; Gruber et al., 2013; Colom and Baucom, 2021).

The extensive body of evidence on the genetic control in root system architecture revealed several quantitative trait loci (QTL) linked to root formation and multiple QTLs controlling single root traits (de Dorlodot et al., 2007; Slovak et al., 2016). Courtois et al. (2009) performed a meta-QTL analysis in rice, gathering 675 QTLs for root parameters, 123 alone for root thickness. Moreover, the ability to change the root phenotype in response to environmental conditions (Nicotra et al., 2010; Lobet et al., 2019) has been extensively documented (Fromm, 2019; Hanslin et al., 2019; Yu et al., 2019; Rao et al., 2021). Gruber et al. (2013) examined the plasticity in the *Arabidopsis thaliana* root system in response to twelve different nutrient deficiencies that resulted in differently shaped root systems with distinct shortcomings. Especially nitrogen and phosphorus tend to have a significant influence on determining root system architecture. These suggest the potential plasticity of root systems in response to habitat and resource availability.

While the pursuit of nutrients strongly influences root formation, soil water content plays a significant role in shaping root systems (Lynch, 1995; Schenk and Jackson, 2002). If primary roots sense a deficit in soil water content, they grow deeper without forming new lateral roots. This drought-influenced stress phenomenon is known as xenobranching (Orman-Ligeza et al., 2018) and is controlled by abscisic acid (Takahashi et al., 2020). Thus, the longer primary root avoids the dry topsoil layers (Comas et al., 2013), where the lack of water can lead to increased heat and even salinity stress due to salt accumulation at the top region. On the other hand, plants growing in an arid climate develop the shallowest primary root as the only available water remains on the surface from rare precipitation events (Schenk and Jackson, 2002).

Furthermore, soil compaction affects the formation of the root system in different ways, and the tolerance provided by plasticity is poorly understood, as it is hard to separate between adaptive mechanisms and ontological processes (Correa et al., 2019). Soil compaction affects the total root length (Pfeifer et al., 2014) and higher root diameter (Merotto Junior and Mundstock, 1999). Hence many interacting factors act in synergy to offer an optimal root architecture suitable for the plant for their respective habitat. However, the relative contribution of genetic predisposition and the local adaption to the environment to the variability or plasticity in root architecture is mainly unknown.

Root plasticity in allocation and physiology is essential to acquiring nitrogen (Hodge, 2004; Freschet et al., 2018). In maize, this plasticity is termed as “steep, cheap, and deep,” which is the optimal root system for acquiring water and nitrogen (Lynch, 2013). On the other hand, phosphorus-deficiency shapes root systems in the opposite direction, where the primary root growth is inhibited and the density of first-order lateral roots increases (Gruber et al., 2013; Niu et al., 2013). Postma et al. (2014) suggested that most plants balance root architecture traits between nitrogen and phosphorus uptake, for instance, lateral root branching density. While lateral roots are essential to foraging nutrients, the control mechanisms are largely unknown (Pelissier et al., 2021). Auxin positively correlates to lateral root initiation, and any disruption to auxin transport decreases lateral root initiations (Boerjan et al., 1995; Reed et al., 1998).

**Figure 1:**
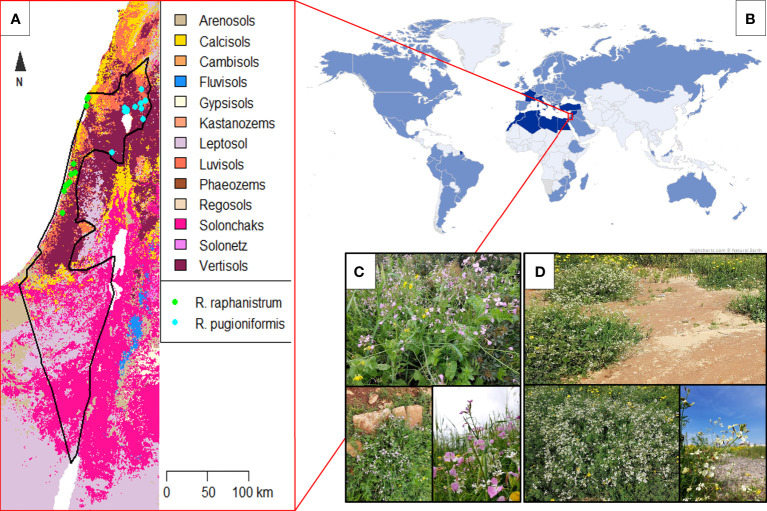
Distinct habit and non-overlapping distribution of *Raphanus raphanistrum* (a worldwide weed) and its relative, *R. pugioniformis* on corresponding soil types in Israel. **(A)** Recorded populations of *R. raphanistrum* (green dots) and *R. pugioniformis* (blue dots) were plotted on a soil map of Israel (Batjes et al., 2020). *R. raphanistrum* occurs mainly on sandy soils of coastal plains (=Calcisol), while *R. pugioniformis* is restricted to mountainous areas in north-eastern Israel on Terra Rossa (=Luvisol) and basaltic (=Vertisol) soils. **(B)** *R. raphanistrum* scattered worldwide (pale blue areas on the world map; (Holm et al., 1997)) from its native distribution area of the Mediterranean basin (highlighted by dark blue; (Ziffer-Berger et al., 2020)). R. pugioniformis is restricted to northern Israel, southern Lebanon, and Syria. **(C)** Representative native population of *R. pugioniformis* at Mt. Gilboa, Israel growing on Terra Rossa soil with high water retention. **(D)** Representative natural populations of *R. raphanistrum* growing on sandy soil (Hamra), which differ in several soil characteristics from Terra Rossa (pH value, nutrient availability, and water retention capacity), near the coastal plain at Tel Aviv, Israel. Worldwide distribution of *R. raphanistrum* is compiled from Holm et al. (1997) and plotted on Miller’s world projection (www.whighcharts.com, based on data from www.naturalearth.com). The soil map of Israel is prepared with the data from Soilgrids (www.soilgrids.org) with 250m resolution.

In this study, we compared the root system architecture of two *Raphanus* species by a non-invasive, semi-automated method. *Raphanus raphanistrum* L. (*Rr*) is a worldwide common weed, causing severe crop loss in agriculture, especially in wheat farming (Holm et al., 1997). Its original distribution is around the Mediterranean basin, along the coastal plains on sandy soil (**Figure 1A**). *Rr* has been spreading to new habitats since the neolithic period, at least in central Europe, probably with the introduction of arable farming. During the Roman empire, it got established in the British Isles and later worldwide (Sampson, 1967; Parsons and Cuthbertson, 2001). Suitable soil is variable for *Rr*; besides avoiding too calcareous, parameters can alter between sandy to clayey, nutrient-rich to poor, and sometimes saline soils are excepted (Mattrick, 1938; Warwick, 1994). According to Parsons and Cuthbertson (2001) and Warwick (1994), *Rr* more often grows on highly fertile acid nitrogenous soil. In contrast, its close relative, *Raphanus pugioniformis* DC. (*Rp*) is endemic to northern Israel, southern Lebanon, and Syria, growing on Terra Rossa and basaltic soil (Ziffer-Berger et al., 2020). This species is not studied as well as *Rr* due to its lower distribution range and not interfering with agriculture. Thus, incomplete *Rp* distribution documentation is probable (Pistrik, 1987).

In this study, we hypothesize that the root architecture of the two species might differ as their natural habitat and soil do not overlap (**Figure 1**). For example, the sandy Hamra soil has a low water-holding capacity in contrast to the clayey, Terra Rossa (Singer, 2007). Furthermore, Terra Rossa overlays hard rocks with a small mantle, limiting rooting depth by an impenetrable lithic soil horizon (Shapiro, 2006). *Rp* might have developed a shallow root system to adapt to such soil characteristics, while *Rr* might have longer main roots to reach deeper soil layers for water. Furthermore, different nutrient foraging strategies may exist as both soils are distinct in several macro and micro-nutrient compositions.

To shed light on the complexity of the root system architecture of both species, we focused on the influence of nutrients and their effects on root formation. Therefore, we recorded multiple variables, which demands a dimensional reduction through principal component analysis, to reveal root structure plasticity’s primary cause and effect in the two species.

Finally, based on the findings, we attempted to expand our understanding of the worldwide spread of *Rr* in contrast to its close relative *Rp*.

## Methods

### Plant material

Seeds of both species were initially collected in Israel between 2016-2017 from at least ten populations spread over a sampling area of more than 12,000 km^2^. 25-30 matured fruits of *R. pugioniformis* were collected around the northern districts of Israel (Golan Heights and Mount Gilboa, see **Table 1**) from wild populations growing on Terra Rossa soil. In contrast, 25-30 fruits of *R. raphanistrum* were collected around the central district of Israel (Tel Aviv, see **Table 1**) along the coastal plain (**Figure 1A**). The seeds were manually released from the pericarp and treated with 50 µM gibberellic acid to break the physiological dormancy. Seeds were germinated in climate chambers (25°C for 8/16 h in light/darkness) and transferred to germination trays after one week. After two weeks, 30-40 individuals per population were randomly selected for mass propagation inside separate net houses in the field station in Israel (Wasserstrom et al., 2022). Matured fruits were harvested on the same day and stored at room temperature before shipping to Osnabrück University.

**Table 1:** Representative collection sites of wild *Raphanus pugioniformis* and *Raphanus raphanistrum* populations in Israel.

Collection side	Latitude	Longitude	Temperature (max. month average)	Temperature (min. month average)	Precipitation (mm year^1^)
*R. pugioniformis*					
Gilboa	32°30′15.95″ N	35°24′43.52″ E	31.3°C	5.8°C	546
Yehudia	32°57′10.01″ N	35°42′23.02″ E	32.3°C	6.3°C	501
Nov	32°50′4.251″ N	35°47′51.28″ E	32°C	5.1°C	476
Arbel	32°49’26.7” N	35°29’54.6” E	31.8°C	7.5°C	503
Salukia	32°58’54.4” N	35°43’53.0” E	32.1°C	5.3°C	551
*R. raphanistrum*					
Ra’anana	32°11′27.80″ N	34°50′45.73″ E	30.5°C	8.2°C	563
Ilanot	32°17′29.91″ N	34°53′55.25″ E	31.1°C	7.7°C	569
Giva’a Haim	32°23′41.13″ N	34°55′54.15″ E	31°C	7.6°C	567
Gesher haZiv	33°02′32.44″ N	35°06′35.81″ E	30.8°C	7.9°C	593
Nes Ziona	31°55’60.0” N	34°47’16.4” E	31°C	7.6°C	539

### Seed germination

The after-ripened seeds were released manually by removing the hard pericarp from randomly chosen fruits. The seeds were washed with distilled water and sterilized in 0.4% (w/v) NaOCl for 10 minutes. After sterilization, the seeds were rinsed twice with distilled water, and 10-20 seeds were transferred to a petri dish lined with moistened filter paper (Macherey-Nagel, MIIV 615, 90 mm) containing freshly prepared 0.1 mg/ml Gibberellic Acid (GA_3_). The Petri dishes were wrapped with parafilm and stored in climate chambers (Sanyo, Versatile Environment Test Chamber, MLR-352) at 25/15°C with a day/night photoperiod of 16/8 h (12000 lux). After germination, the seedlings remained in the Petri dishes for five to seven days to maximize seedling survival in the downstream experiments. Only seedlings with healthy radicle and fully developed cotyledons were selected.

### Controlled root growth in natural habitat

**Figure 2:**
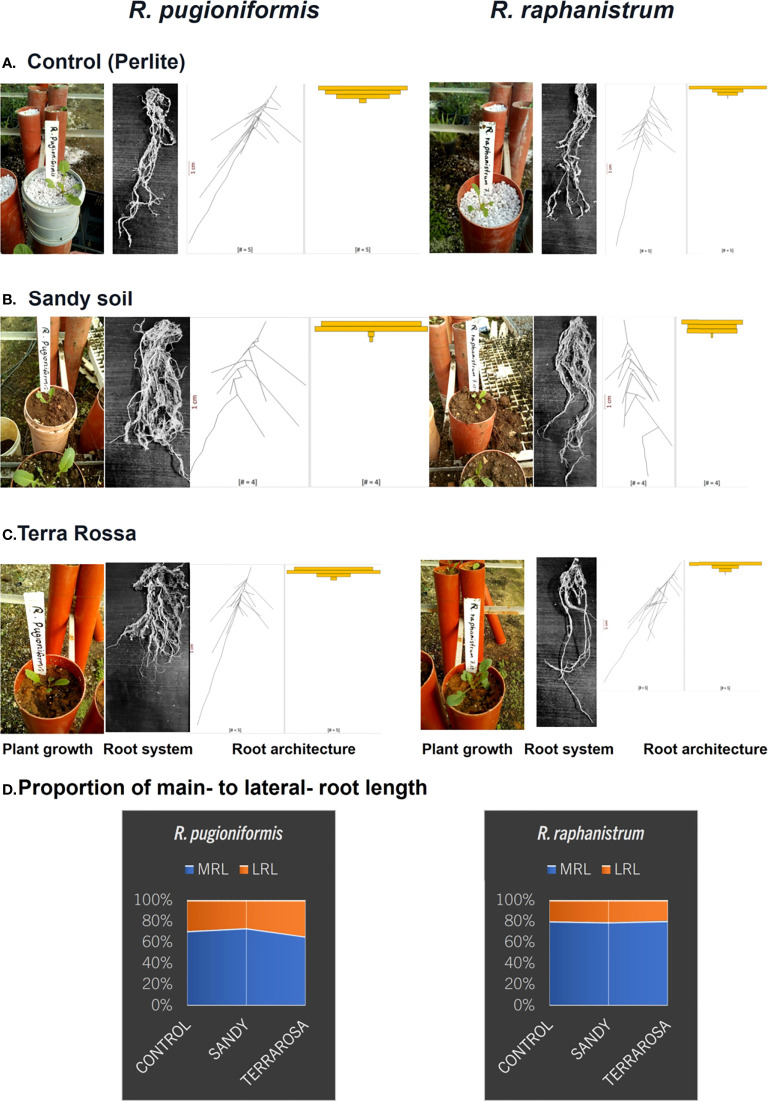
Characteristic difference between main and lateral root lengths of *R. pugioniformis* and *R. raphanistrum* growing on native and non-native soils. *R. pugioniformis* requires significant modulation of main- to lateral-root length proportion on different soils **(A–C)**, while the general root architecture of *R. raphanistrum* remained unaffected **(D)**. The two species were potted in three different soils, control **(A)**, Hamra, native soil of *Rr* **(B)**, and Terra Rossa, native soil of *Rp* **(C)**. The plants were excavated at the start of bolting (78 days after germination), roots were rinsed from substrate, disentangled, photographed, and analysed *via* EZ-Rhizo II (see methods section for details). Next to the representative photographs of rinsed, disentangled root system, digitized root characters are presented as alpha blends, and the spread of Lateral Root Length (LRL) computed by Root-VIS II. The proportion of main- to lateral- root length **(D)** in all three soils were calculated from the digitized root parameters.

**Figure 3:**
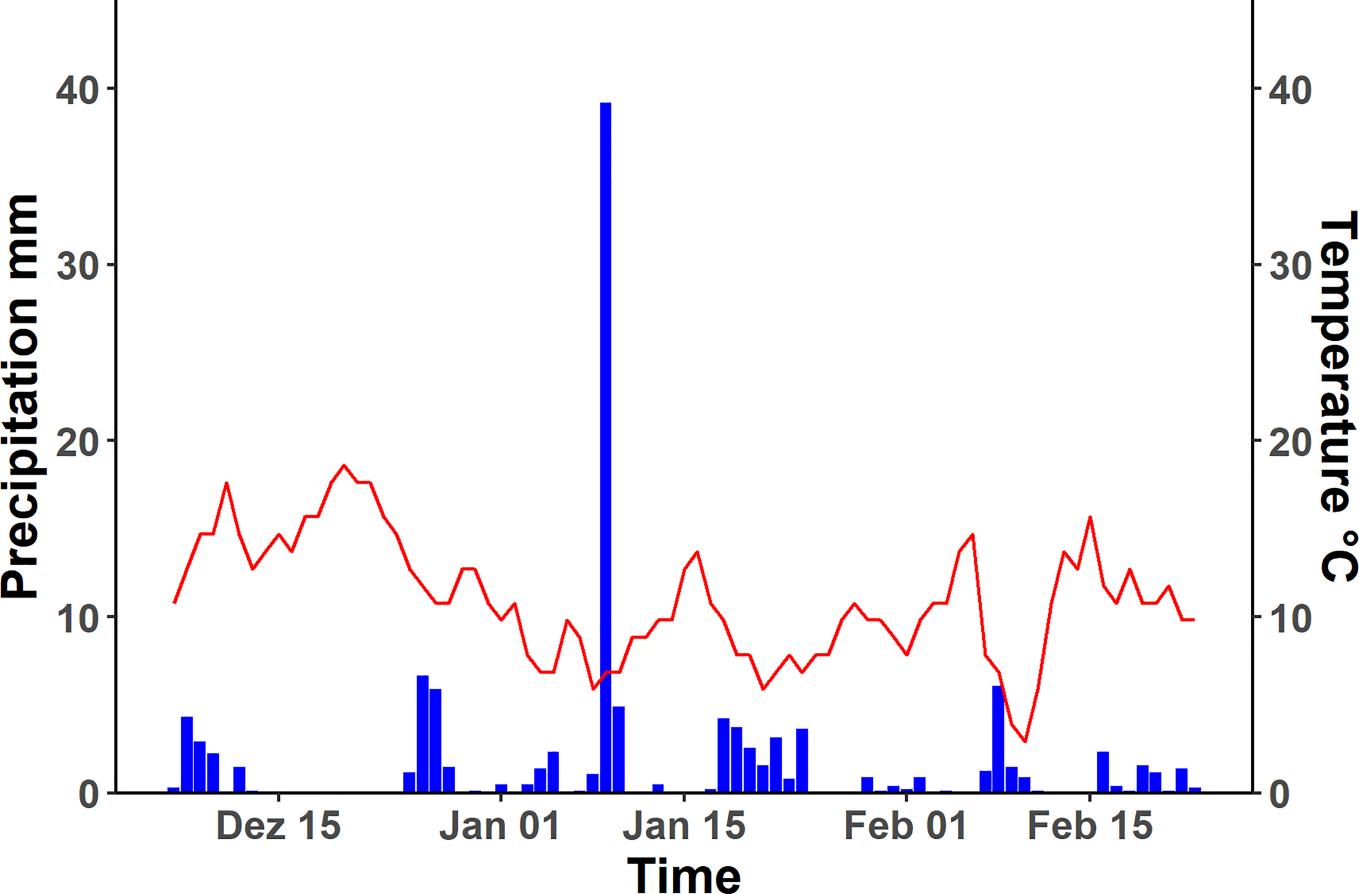
Average daily temperature and precipitation of Tulkarm over the growth period of plants used for experiment. Left y-axis shows the precipitation in millimetre, indicated by the blue bars, on the right side the temperature is given in degree Celsius, plotted in the graph as red line. The growth started at 07.12.2019 and ended with the harvest at 23.02.2020.

The seedlings were transferred to cylindrical pots (diameter, 11 cm; length 1m) filled with the natural unsieved soils from *Rr* (Hamra) and *Rp* (Terra Rosa); perlite medium (particle size 5 ± 2 mm) was chosen as control (**Figure 2**). Five pots were filled per substrate for each species, leading to 30 pots total (5 plants x 2 species x 3 substrates = 30 replicates). The transplanted seedlings were allowed to grow under unroofed net houses at the ARID from 07.12.2019 until 23.02.2020 and were exposed to the local weather parameters. The mean temperature during the experiment was 11°C, mean precipitation was 1.48 mm per day, with a maximum of 39.18 mm and a total of 116.57 mm. The most extended rainless period was eleven days; otherwise, they were no longer than four days (**Figure 3**). Roots were harvested after bolting the rosette plants after 78 ± 5 days. Every root system was photographed under uniform light conditions after the root system was rinsed thoroughly to remove soil particles, and the major roots were disentangled for efficient digitization.

### Controlled root growth

**Figure 4:**
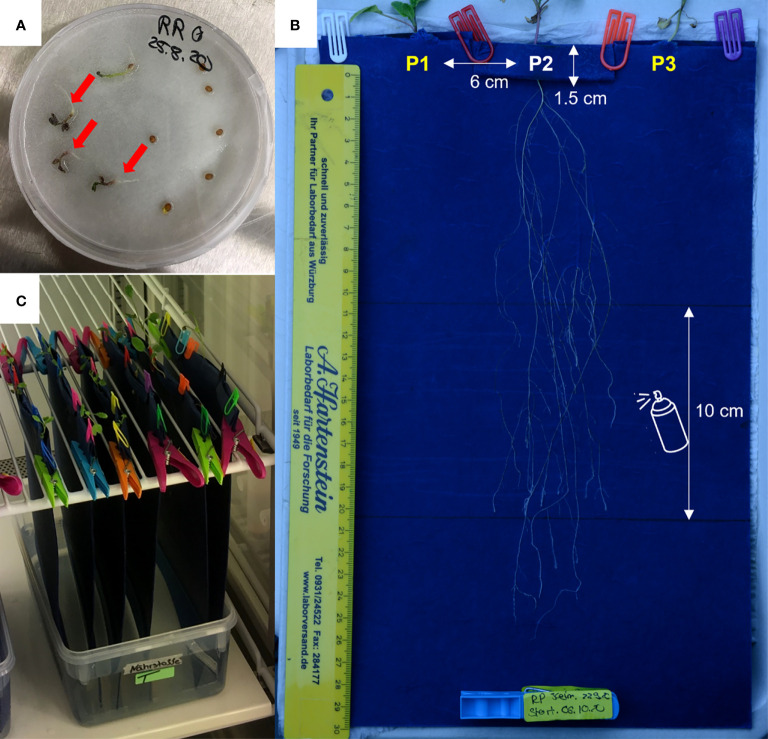
Experimental setup to mimic root growth in surrogate media for soil types and simultaneous, semi-automated root growth analysis. **(A)** Seedlings of *R. raphanistrum* and *R. pugioniformis* were germinated in petri dishes on moistened filter paper, until the radicles were clearly visible and cotyledons were fully developed (red arrow). **(B)** The seedlings were transferred to the root growth experimental set up. Three pockets of 1.5 cm depth were folded on the upper edge, with a hole at the bottom, to insert the radicle through. The distance between the seedlings was 6 cm. Two seedlings were placed on one side of the paper near the edges, and a third seedling was placed in the middle on another side (visible in the figure). The middle section of 10 cm was sprayed with soil surrogate every other day. **(C)** All the experimental setup were hung vertically in a climate chamber, with the lower edge touching a reservoir of distilled water for continuous capillary water uptake. Altogether 27 seedlings per species were analysed for root architecture (3 soil surrogates X 3 seedlings X 3 setups).

Seedlings were transferred to custom-designed folded pockets of seed germination paper (Sartorial Stedim Biotech, 200 x 350 mm, **Figure 4**). Three pockets were made on the folded upper edge of each paper each 3 cm apart from the edge, and with a depth of 1.5 cm. The structural design of the two flanking pockets on one side and the central pocket on the reverse side allowed synchronous growth of the three seedlings on the same paper. The protruding radicle of a germinated seed was carefully inserted through the small hole at the bottom of each pocket. The pockets were subsequently secured with plastic clips to ensure the attachment of the radicles to the paper. Three similar setups were prepared for each species, leading to nine replicates per species and soil surrogates (3 plants x 3 setups x 3 soil surrogates = 27 replicates per species). The setups were secured vertically in climate chambers under the same conditions described before for germination. Due to the vertical position, the roots could follow natural gravitropism (**Figure 4**).

The plants had constant access to capillary water as the lower ends of the germination papers were dipped in distilled water stored in a 2500 ml box. The supplemental nutrient solutions (1 ml), corresponding to the soil surrogates, were applied by spraying on each side of the root growth set up every other day at 10 cm below the pockets and 10 cm away from the edge of the paper but not directly on the growing roots. These soil surrogates were used to mimic Hamra and Terra Rossa; the third solution was used as a control and contained a modified version of the Hoagland and Knop medium (HiMedia Laboratories, 2017; **Table 2**).

**Table 2:** Composition of soil surrogate root growth media to mimic Hamra and Terra Rossa soils and a control.

Soil surrogate	Component	Stock concentration (mole)	Final concentration (millimole)	Volume for 1000 ml (millilitre)
Minimal Media (Control)	CaCl2	0.125	0.5	4
	MgSO4	0.25	0.25	1
	KNO3	1	1	1
	KH2PO4	0.2	0.5	2.5
	Fe(III)Na2-EDTA	0.0425	0.0425	1
	Tris	0.1	0.5	5
	MES	0.2	2.8	14
	Micronutrient stock solution	1000 x	1 x	1
	H2O			970.5
Hamra	CaCl2	0.125	0.625	5
	MgSO4	0.25	0.25	1
	KNO3	1	1.2	1.2
	KH2PO4	0.2	0.8	4
	Fe(III)Na2-EDTA	0.0425	0.085	2
	Tris	0.1	0.5	5
	MES	0.2	2.8	14
	Micronutrient stock solution	1000 x	1 x	1
	H2O			966.8
Terra Rossa	CaCl2	0.125	0.6875	5.5
	MgSO4	0.25	0.175	0.7
	KNO3	1	1.5	1.5
	KH2PO4	0.2	0.7	3.5
	Fe(III)Na2-EDTA	0.0425	0.2975	7
	Tris	0.1	0.5	5
	MES	0.2	2.8	14
	Micronutrient stock solution	1000 x	1 x	1
	H2O			961.8

The pH was adjusted with MES or Tris to a pH of 6.3, respectively to 7.5 for Terra Rossa, before the volume was filled up to 1000 ml.

### Root growth monitoring

The root growth setups were briefly removed from the growth chamber on the 7^th^, 14^th^, and 21^st^ days after the seedlings were transferred on the germination paper to take digital images. Pictures (.jpeg) were taken with uniform non-reflecting light conditions (with two soft lightboxes in a dark room) with a digital camera (Nikon D7100, lens: Sigmar 17-70 mm 1:2.8-4DC, 72) with a constant setting of f/13, ISO 200, shutter speed 1/25 sec to avoid any variable in image acquisition and its effect on further downstream processing.

### Semi-automated digital analysis of root architecture

**Figure 5:**
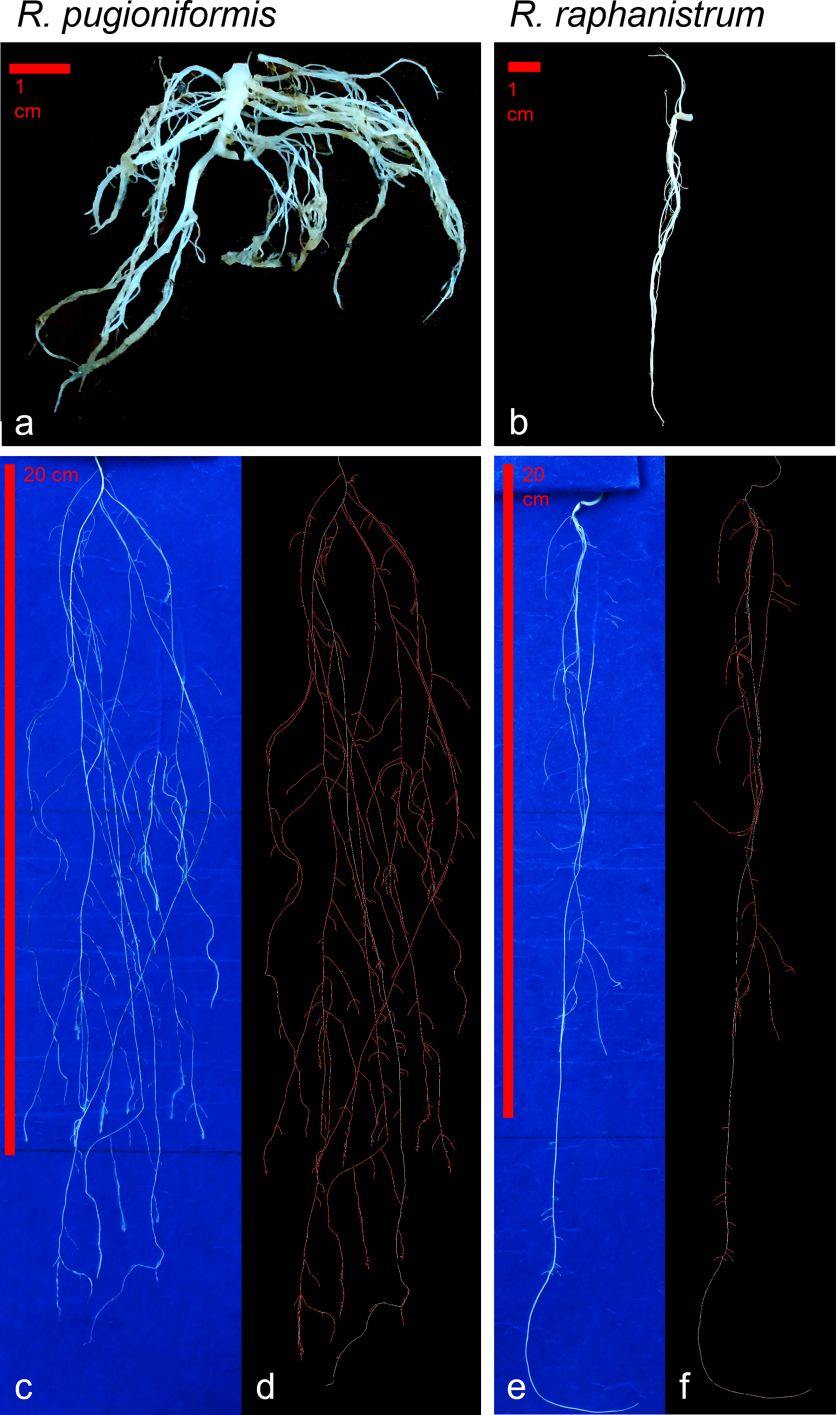
Root architecture differs between *R. pugioniformis* and *R. raphanistrum*. **(A)** Representative root architecture of *R. pugioniformis* grown on Terra Rossa soil, showing a short main root with elaborate lateral root system. **(B)** *R. raphanistrum* grown on Hamra formed a long main root and few lateral roots. The plants were harvested after bolting (approximately 70-80 days after germination) following growth in natural conditions with individual pots in Palestine (see **Figure 2**). Representative root architectures of *R. pugioniformis* **(C)** and *R. raphanistrum* **(E)** after 21 days of controlled growth on modified root growth media (Hoagland & Knop medium), mimicking native soil nutrient composition of Terra Rossa and Hamra for *R. pugioniformis* and *R. raphanistrum*, respectively. Digital profiling of the corresponding roots after analysis with EZ-Rhizo II [*R. pugioniformis*, **(D)**; *R. raphanistrum*, **(F)**], which enables quantitative analysis of root architecture and growth kinetics. The main root is traced as white line, while all the lateral roots are drawn in red.

**Figure 6:**
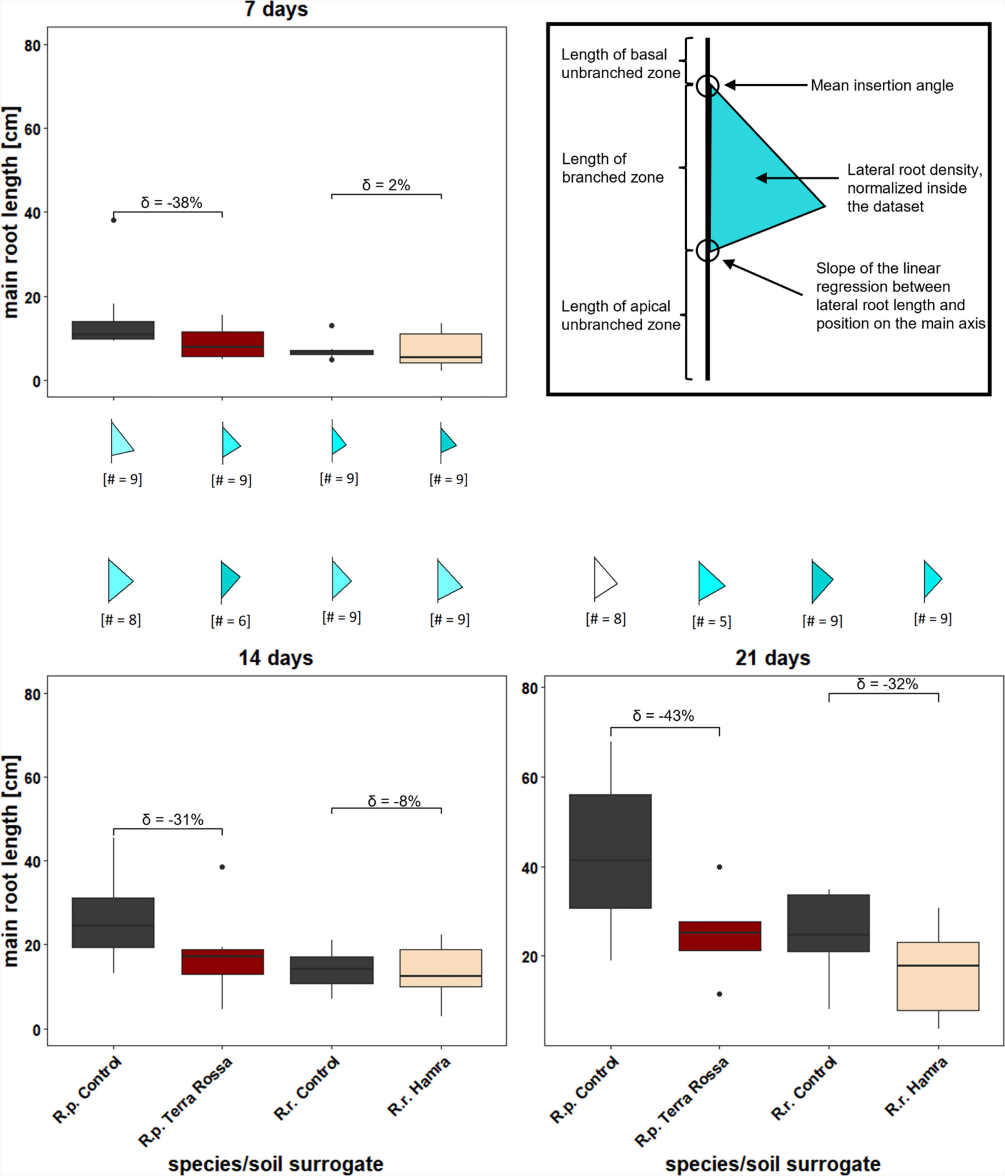
Different root growth kinetics in diverse surrogates of soil types indicates a faster root growth on control, than on the species natural soils. Native soil types of both species, *R. pugioniformis Rp*, Terra Rossa) and *R. raphanistrum* (*Rr*, Hamra), along with a control, were mimicked by specific nutrient solutions (details see M&M). Root growth was recorded after one, two, and three weeks for semi-automated root architecture analyses. The percentage difference of main root length between control and native soil of the respective species are represented as delta (δ) above the box plots. The corresponding flag plots represent summary of different root system architecture parameters computed via EZ-Rhizo II. A typical "flag plot" (upper right panel with bold outline) represents mean insertion angle of lateral roots, displayed by the upper angle from "flagpole" and "flag", length of different root zones, departed by the triangle into basal unbranched zone, length of branched zone and length of apical unbranched zone. Furthermore, lateral root density is indicated by the color intensity within the triangle, ranging from white (low) to dark turquoise (high root density) and the slope of the linear regression, displayed by the lower angle (details see M&M). *Rp* = *R. pugioniformis*, *Rr* = *R. raphanistrum*.

A semi-automated root architecture analysis was carried out with EZ-Rhizo II version 2.5.0.1 (Shahzad et al., 2018). Briefly, the raw images of the root system were converted to skeletonize the root outlines for automated node and branch detection. Manual curation of the raw images was necessary for specific images where natural overlapping of the roots could impact the analysis (**Figure 5**). Measured variables were the main root length, main root angle, main root vector (the straight-line distance between base and apex (Shahzad et al., 2018)), total root length, the number of lateral roots and the total number of lateral roots. L ateral root length was calculated by substracting the main root length from the total root length. Collected data was stored as a.xml file and transferred to a.csv table for further analysis. “Flag plots” were generated with Root-VIS II (version 2.5.0.1), summarizing several root system architecture (RSA) parameters in one figure (**Figure 6**). The “flagpole” is divided by a triangle into three parts, where the upper part represents the root length of the basal unbranched zone (**Figure 6**). The middle part is defined as the branched zone, the zone of lateral root growth bordered by the contact points of the triangle. The lower part represents the apical unbranched zone of the main root. The upper angle, where the triangular “flag” and the “flagpole” meet, indicates the mean insertion angle of the lateral roots. The lower angle shows the linear regression slope between lateral root length and its position on the main axis. The color within the triangle represents the density of lateral roots. The darker the color, the denser the lateral roots compared to the other samples simultaneously. For the comparison, the samples were normalized to a value between 0 and 1 (Shahzad et al., 2018).

Furthermore, the root architecture of the potted plants was visualized using Root-VIS II. The first visualization, alpha blends, is a digital reconstruction of all roots combined per treatment, so the general root structure is inferred (**Figure 2**). A lateral root length (LRL) plot was also calculated to represent the distribution of lateral roots along the main root (**Figure 2**).

### Statistical analyses

Statistical analysis was performed with R version 4.0.5 (R Core Team, 2021). Every dataset was tested for normal distribution *via* Shapiro-Wilk normality test (‘shapiro_test’ function, package ‘rstatix’, (Kassambra, 2021), followed by an ANOVA (‘anova_test’ function, package ‘rstatix’, (Kassambra, 2021) or, if not normally distributed, with a Scheirer-Ray-Hare test (‘scheirerRayHare’ function, package ‘rcompanion’, (Mangiafico, 2021). For normally distributed data TukeyHSD (‘TukeyHSD function, package ‘stat’, (R Core Team, 2021) was chosen as a *post-hoc* test, otherwise, the Dunn test [function: ‘dunnTest’, package: ‘FSA’, (Wheeler and Dinno, 2021)] was performed. We primarily focused on the main root length, as this parameter revealed the most information regarding the depth of root growth of the species (Freschet et al., 2021). The boxplots were created with the R package ‘ggplot2’ (Wickham, 2021). The delta value was calculated as a percentage difference of the main root length within the species between the control and native soil. The control was assumed to be 100%, and the resulting difference in the soil is expressed with delta (**Figure 6**).

**Figure 7:**
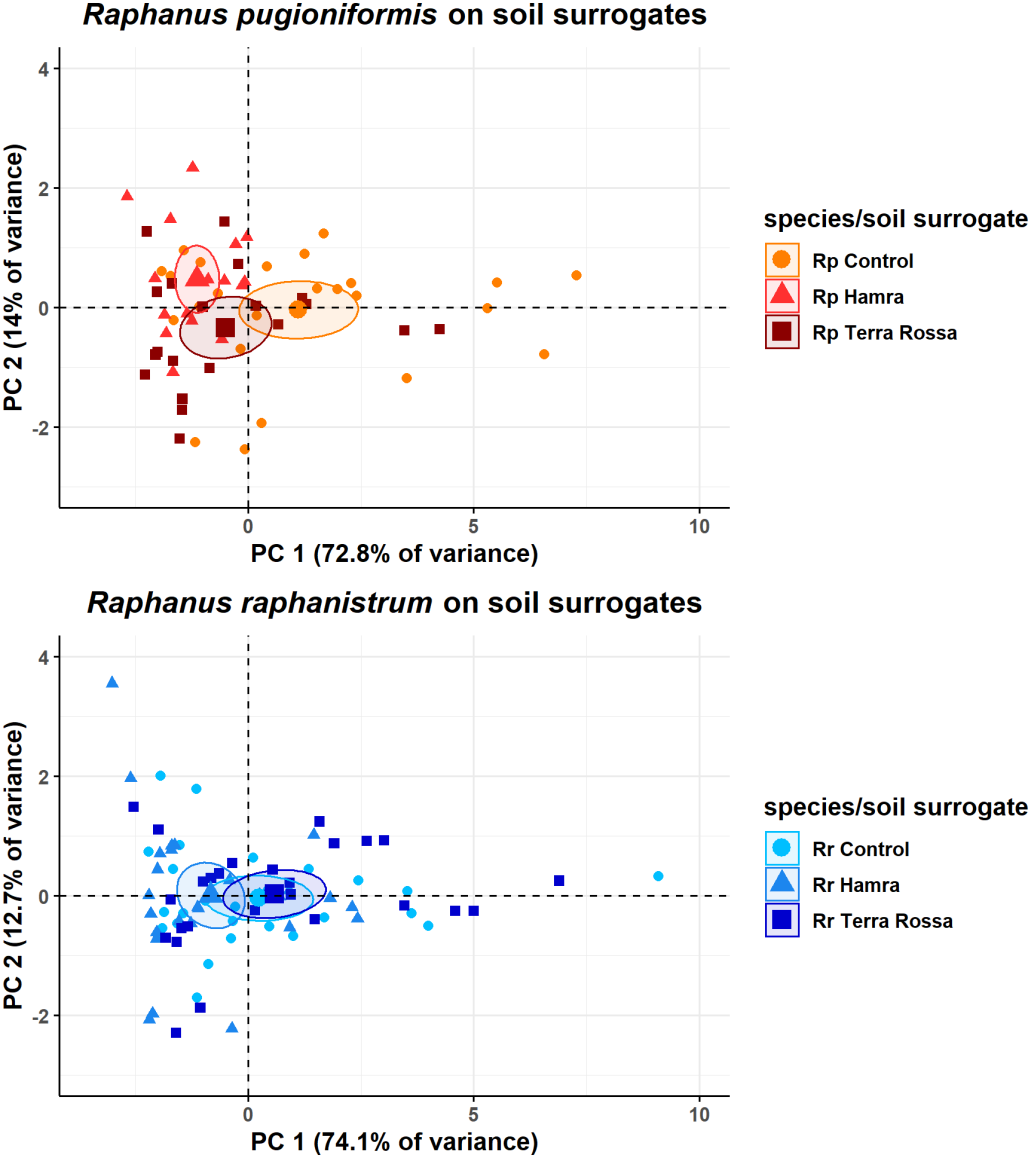
Dimensional reduction of seven root architecture parameters revealed greater modulation of lateral root length and number is required by *R. pugioniformis* than by *R. raphanistrum* to adapt to diverse soil types. PCA analysis of seven root parameters from *R. pugioniformis* and *R. raphanistrum* grown on three different soil surrogates over three weeks (126 samples in total). Soil surrogates represent nutrient solutions, mimicking the natural soils of *R. raphanistrum* and *R. pugioniformis*, i.e., Hamra and Terra Rossa, the third solution is a control. Principal component 1 separates the groups by length of different root types and the number of lateral roots, component 2 mainly by main root angle, together they include 85% of the total information content derived from the measured variables (main root length, main root vector, main root angle, total root length, lateral roots on main root, total number of lateral roots and lateral root length). The ellipses show the 95% confidence interval.

Furthermore, principal component analyses (PCA) were performed [R package ‘factoextra’, (Kassambra and Mundt, 2020)] with 61 values from *Rp* and 81 values from *Rr*, grown with two soil surrogates and on control over three weeks (**Figure 7**). The parameters used for calculation were main root length, main root vector, main root angle, total root length, lateral roots on the main root, total number of lateral roots, and lateral root length. To asure the suitability of the data for a PCA, the Kaiser-Meyer-Olkin criterion [R package ‘psych’, (Revelle, 2022)] as well as a Bartletts test of sphericity was performed beforehand [R package ‘psych’ (Revelle, 2022)].

## Results

### Distinct root morphology of *R. pugioniformis* and *R. raphanistrum*

The root architecture of *Rp* is widely branched out and shallow (**Figures 2**, **5**). Such a root structure is evident from the individual plant grown in its natural soil Terra Rossa, which produced 68 lateral roots, with a total root length of 264.2 cm, but only 20 cm contributing to the main root length. The main root angle was 21.3°. In contrast, the *Rr* plant, grown in Hamra, produced 17 lateral roots, with a total root length of 103.6 cm, showing a root architecture growing more into deep soil (**Figure 5B**), with the main root contributing 35.3 cm of it. While the main root of the *Rp* contributed only 7.6% of the total root length, 34% of the root length in *Rr* was contributed by the main root with a steeper angle (8.2°) than *Rp*.

### Plasticity of root architecture on different soils

The roots that grew in perlite and the natural soils of *Rr* and *Rp* demonstrated a clear difference in root architecture. While *Rp* formed a shallow expanded RSA on its natural Terra Rossa, *Rr* grew deeper and less branched roots in every soil. The proportion of main- to lateral- root length stayed equal for *Rr* at around 80:20, and *Rp* developed more lateral roots on Terra Rossa (60:40) in comparison to the other treatments (70:30) (**Figure 2**).

### Distinct insertion angle for lateral roots for deep and shallow soil penetration

Generally, plants on controls and native soil surrogates showed an unbranched apical zone after the first week, which was not evident in the following weeks according to the flag plots (**Figure 6**). Furthermore, a steeper insertion angle for the lateral roots of *Rr* was observed, implying it has a deep penetrating root system compared to *Rp*.

### Significant differences in root architecture between the species

Steady root growth was observed with each treatment. The box plots (**Figure 6**) displayed a faster growth of main roots in *R. pugioniformis* than in *R. raphanistrum* over the whole observation period and in both soil surrogates. Furthermore, both species performed better on the control soil surrogate than on the one representing the natural soil of the individual species. The difference between the control and the native soil surrogate, expressed with delta, was not very high for *Rr* in the first two weeks, laying at maximal -8% (SE ± 4.5 cm). Delta increased in the third week to a -32% (SE ± 9.3 cm) difference compared to the control. In contrast, *Rp* showed a huge delta, never lower than -31% (SE ± 12.8 cm) and with a maximum of -43% (SE ± 22 cm) after the third week.

### Major root growth parameters of *R. raphanistrum* are unperturbed in different soil surrogates, but not for *R. pugioniformis*

First, the data was tested for adequacy for a PCA by Kaiser-Meyer-Olkin criterion, resulting in an overall measure of sampling adequacy of 0.79. As a value between 0.8 and 0.7 is categorized as middling by Kaiser and Rice (1974), also Bartletts test of sphericity was performed. The calculated p-value 6.68e-152 is indicating the data is appropriate for a PCA. The performed PCAs revealed (**Figure 7**) that the first two calculated principal components (PCs) explain 87% of the variance for *Rp* (**Appendix 1**) and 86.8% for *Rr* (**Appendix 3**). The first PC of *Rp* contains 72.8% variance. The loading of all variables for PC1 is evenly distributed between 13% and 16%, except the main root angle with only 3.1% (**Appendix 2**). Conversely, for the second PC, which contained 14% variance, the main root angle had the highest loading with 83%. The results of *Rp* show a separation of all soil surrogates, primarily influenced by PC1.

For *Rr*, the overall measure of sampling adequacy was also middling with 0.73, the appropriation for a PCA was indicated by a p-value of 8.67e-235 after Bartletts test of sphericity. PC1 contains 74.1% variance, and the loadings are equally distributed between 15% and 16%, except for the main root angle, which has a loading of 6.2% (**Appendix 4**). PC2 explains 12.7% of the variance, and the highest loading is 72.5% for the main root angle. The three resulting groups differ slightly on PC1, but no clear separation in groups can be observed. On the y-axis, the medians all show a similar value. Comparing the remaining PCs was neglected, as their eigenvalues were below one, resulting in less information than a variable itself is giving.

Opposing the results of both species, *Rr* shows less alteration in all measured root parameters between the three soil surrogates than *Rp*. These result in a clear separation of independent groups for each soil surrogate for *Rp*, which is not valid for *Rr*.

## Discussion

The seedlings of two *Raphanus* species (*Rr* and *Rp*) grown either in native soils or controlled conditions in the lab revealed apparent differences in their root system architecture (RSA). *Rr* developed long main roots and a steep main root angle in both soil types and perlite control pots (**Figure 2**). On the other hand, *Rp* developed a shallow, branched-out root system at all three treatments. The distinct RSA of *Rr* is in congruence with the previous observations of long and deep penetrating taproots (Cheam and Code, 1995; Holm et al., 1997) and the steep insertion angle typical of the deep root system (Uga et al., 2015). Moreover, the seedlings grown in a controlled environment with soil surrogates, mimicking the two native soil compositions, corroborated with the RSA of *Rr* and *Rp* grown in natural conditions. While the continuous data collection and analysis of RSA in field-grown plants are technically challenging, the customized and semi-automated root growth analysis in controlled climate chambers provided a reliable proxy *via* the appropriate soil surrogates (**Figure 4**).

One of the most significant advantages of controlled root growth analysis is the possibility of simultaneous and continuous root growth monitoring in several individuals. Furthermore, the root growth dynamics can be analyzed with the same individual plant, minimizing the biological and technical variability in conducting a similar experiment with several plants *in situ*. The primary drawbacks of root architecture analysis in plants grown *in situ* are the difficulties associated with excavating and processing the root system before the study. Typically, manual excavation of roots in native soils causes unavoidable damage and loss of root architecture during rinsing soil from the root and disentangling lateral roots of higher orders for digital root structure analyses. Therefore, gathered trait values, evaluated in such a manner, typically have a specific error (Pérez-Harguindeguy et al., 2013). Nevertheless, the field data provides essential insights into the “real” environment, allowing to compare and customize experimental design, to get as close as possible to mimicking the field conditions. Hence, in a carefully designed controlled experiment with seedlings grown on paper, the entire root system could be assessed non-invasive and dynamic way, which offered flexibility to manipulate and observe the effect of nutrients on root architecture.

Our experiment focused on the different nutrient compositions in Hamra and Terra Rossa. Nitrogen (N) and phosphorus (P) play a significant role in root system modulation (Rao et al., 2021; Lynch, 2022). The high mobility of N is particularly significant in porous, sandy soils, like Hamra, which do not hold enough nutrients at the soil surface (Singer, 2007). The long taproot system of *Rr* corresponds to the RSAs found in plants foraging for nitrogen in N-deficient soils (Gruber et al., 2013; Lynch, 2013; Koevoets et al., 2016). Shortage of N is also positively correlated with higher deep root fractions in plants and the percentage of roots growing below 20 cm (Freschet et al., 2018). This is evident from the study as we observed *Rr* had relatively more roots in the lower parts of the nitrogen-poor perlite and Hamra, while in Terra Rossa, more roots can be found in the upper regions (**Figure 2**). It should be mentioned that the plants in our setup did not grow roots much deeper than 20 cm, but even on a smaller scale, the observation still seems to fit a projected extrapolation. The root system of *Rp* shows an opposite pattern by forming many shallow lateral roots. This root system architecture has also been monitored in other species, often indicating P-deficient soils (Gruber et al., 2013; Niu et al., 2013). Due to its low mobility, topsoil hurbours high P *via* the decomposition of organic material (Lynch and Brown, 2001). In the Terra Rossa, plant-available P was found to be less than five ppm, while other micro-and macronutrients are well supplied, which makes the acquisition of P problematic in Terra Rossa, as high amounts of iron probably bind most of it. (Singer, 2007).

All these aspects explain how the two related *Raphanus* species fit in their native habitat. However, that does not address the global distribution of *Rr* on various soils (Pistrik, 1987), while *Rp* is an endemic species in the East Mediterranean.

This study primarily focused on the root architecture of the two species in response to their native habitat. The results showed that only a handful of root parameters were insufficient to address the overarching adaptive evolution of the two diverse RSAs of the two species. Therefore, we performed a multi-dimensional reduction of seven root parameters *via* PCA, demonstrating that the response to all three soil surrogates was not dimensionally resolved for *Rr*, but they were distinct for *Rp*. In other words, this indicates that *Rr* only needs to modulate root parameters marginally to adapt to different soils, while *Rp* needs to make significant changes, costing valuable resources. The PCA analyses were restricted within species to resolve the influence of soil composition (fixed variable) on root parameters (dependent variables). The rationale behind the restriction is the diverse phenology and root-shoot dimensions of the two species that do not allow a simple linear normalization of the root parameters between the two species, which would compromise the resolution of data and the study’s unbiased outcome. In addition, limited nutrient resources often lead to a trade-off between covering a vast space with roots to get nutrients and placing them precisely at nutrient patches (Campbell et al., 1991). Finally, the broad spectrum of soils where *Rr* is found worldwide, reaching from the sand over loam to clay, being saline or nitrogen-rich, and on chalky or acidic soils, can support this hypothesis (Mattrick, 1938; Warwick, 1994; Holm et al., 1997).

Both species, *Rr* and *Rp*, possess a root system adapted to their natural habitat, partly shaped by nutrient availability within the soil. Furthermore, they show adaptability to different nutrient compositions, where *Rr* needs to modify fewer root growth parameters to accomplish a satisfying growth compared to *Rp*. This resource-saving root formation probably enhances the successful establishment, survival, and distribution of *R. raphanistrum*.

## Data availability statement

The raw data supporting the conclusions of this article will be made available by the authors, without undue reservation.

## Author contributions

SB and KM conceived and designed this study. Plant material was collected by OB and JZ-B. Data collection was done by SB, FG and FP. NK organized the database and performed the statistical analysis. The paper was written by SB and NK, all authors contributed to the article and approved the submitted version.

## Funding

This study was supported by the German Research Foundation’s (DFG) trilateral program (Grant no. MU-1137/16-2)

## Acknowledgments

We thank Jens Varnskühler for support in statistical questions, and Katharina Kleemeier for plant propagation.

## Conflict of interest

The authors declare that the research was conducted in the absence of any commercial or financial relationships that could be construed as a potential conflict of interest.

## Publisher’s note

All claims expressed in this article are solely those of the authors and do not necessarily represent those of their affiliated organizations, or those of the publisher, the editors and the reviewers. Any product that may be evaluated in this article, or claim that may be made by its manufacturer, is not guaranteed or endorsed by the publisher.
