# Supplementary material for: ‘Root of all success’: Plasticity in root architecture of invasive wild radish for adaptive benefit
Source: Front Plant Sci. 2022 Nov 16;13:1035089. doi: 10.3389/fpls.2022.1035089 (PMC9709435; doi:10.3389/fpls.2022.1035089)
Supplement: Supplementary file 1 [file Table_1.docx]

Supplementary Material

# Supplementary Data

Appendix 1: Importance of components of the PCA analysis for *Raphanus pugioniformis*:

|  | PC1 | PC2 | PC3 | PC4 | PC5 | PC6 | PC7 |
| --- | --- | --- | --- | --- | --- | --- | --- |
| Standard deviation | 2.2578 | 0.9881 | 0.74046 | 0.57316 | 0.20271 | 0.0909 | 1.816e-16 |
| Proportion of Variance | 0.7282 | 0.1395 | 0.07833 | 0.04693 | 0.00587 | 0.00118 | 0 |
| Cumulative Proportion | 0.7282 | 0.8677 | 0.94602 | 0.99295 | 0.99882 | 1 | 1 |

Appendix 2: Loadings of principal components of the PCA analysis for *Raphanus pugioniformis*:

|  | PC1 | PC2 | PC3 | PC4 | PC5 | PC6 | PC7 |
| --- | --- | --- | --- | --- | --- | --- | --- |
| Main root length | 16.715518 | 2.0650538 | 10.317566 | 19.7275069 | 0.3276581 | 38.945019 | 5.96913e+00 |
| Main root vector | 16.561215 | 6.0504053 | 7.51928 | 22.1596467 | 19.2723509 | 30.90069 | 7.887511e-15 |
| Main root angle | 3.143409 | 82.9596403 | 1.796558 | 0.4831186 | 1.1454654 | 1.498314 | 8.989443e-16 |
| Total root length | 16.998771 | 2.9379263 | 13.397397 | 10.66326 | 15.6041801 | 4.351022 | 4.970206e+01 |
| Lateral roots on main root | 13.624301 | 0.7576115 | 40.330655 | 8.4330422 | 16.9534547 | 4.011684 | 1.241123e-14 |
| Nr. Of lateral roots in total | 16.148374 | 2.2133915 | 13.006519 | 23.9212113 | 29.2453939 | 10.170673 | 2.609838e-14 |
| Lateral root length | 16.808411 | 3.0159713 | 13.632023 | 14.6122143 | 17.4514968 | 10.122597 | 4.432881e+01 |

Appendix 3: Importance of components of the PCA analysis for *Raphanus raphanistrum*:

|  | PC1 | PC2 | PC3 | PC4 | PC5 | PC6 | PC7 |
| --- | --- | --- | --- | --- | --- | --- | --- |
| Standard deviation | 2.2786 | 0.9441 | 0.75712 | 0.52367 | 0.198 | 0.17309 | 1.141e-16 |
| Proportion of Variance | 0.7417 | 0.1273 | 0.08189 | 0.03918 | 0.0056 | 0.00428 | 0 |
| Cumulative Proportion | 0.7417 | 0.869 | 0.95094 | 0.99012 | 0.9957 | 1 | 1 |

Appendix 4: Loadings of principal components of the PCA analysis for *Raphanus raphanistrum*:

|  | PC1 | PC2 | PC3 | PC4 | PC5 | PC6 | PC7 |
| --- | --- | --- | --- | --- | --- | --- | --- |
| Main root length | 15.27962 | 7.064351 | 23.68818775 | 10.4953485 | 21.116417 | 24.5368088 | 1.266718e+01 |
| Main root vector | 15.269628 | 8.014573 | 24.10631256 | 7.3458872 | 14.8084717 | 30.7708457 | 3.522261e-14 |
| Main root angle | 6.233488 | 71.470232 | 3.46695934 | 0.6398524 | 0.4378564 | 0.7267609 | 4.402827e-15 |
| Total root length | 16.516937 | 5.328304 | 0.09249228 | 19.8285025 | 0.6125297 | 7.7975514 | 4.877103e+01 |
| Lateral roots on main root | 15.447491 | 1.238690 | 17.64862906 | 23.4951925 | 26.5200869 | 15.2682993 | 9.90636e-15 |
| Nr. of lateral roots in total | 15.382251 | 2.465451 | 23.33305965 | 9.6695032 | 28.7934045 | 19.0978852 | 3.522261e-15 |
| Lateral root length | 15.870585 | 4.418401 | 7.66435935 | 28.5257136 | 7.711234 | 1.8018487 | 3.85618e+01 |
